# Self-Aligned Emission of Distributed Feedback Lasers on Optical Fiber Sidewall

**DOI:** 10.3390/nano11092381

**Published:** 2021-09-13

**Authors:** Tianrui Zhai, Xiaojie Ma, Liang Han, Shuai Zhang, Kun Ge, Yanan Xu, Zhiyang Xu, Libin Cui

**Affiliations:** Faculty of Science, College of Physics and Optoelectronics, Beijing University of Technology, Beijing 100124, China; xiaojiema@emails.bjut.edu.cn (X.M.); hanliang@emails.bjut.edu.cn (L.H.); 13844225221@163.com (S.Z.); GEKUN@emails.bjut.edu.cn (K.G.); 18844169792@163.com (Y.X.); xu.zhiyang@hotmail.com (Z.X.)

**Keywords:** distributed feedback lasers, colloidal quantum dots, self-aligned emission, optical fiber, mode coupling

## Abstract

This article assembles a distributed feedback (DFB) cavity on the sidewalls of the optical fiber by using very simple fabrication techniques including two-beam interference lithography and dip-coating. The DFB laser structure comprises graduated gratings on the optical fiber sidewalls which are covered with a layer of colloidal quantum dots. Directional DFB lasing is observed from the fiber facet due to the coupling effect between the grating and the optical fiber. The directional lasing from the optical fiber facet exhibits a small solid divergence angle as compared to the conventional laser. It can be attributed to the two-dimensional light confinement in the fiber waveguide. An analytical approach based on the Bragg condition and the coupled-wave theory was developed to explain the characteristics of the laser device. The intensity of the output coupled laser is tuned by the coupling coefficient, which is determined by the angle between the grating vector and the fiber axis. These results afford opportunities to integrate different DFB lasers on the same optical fiber sidewall, achieving multi-wavelength self-aligned DFB lasers for a directional emission. The proposed technique may provide an alternative to integrating DFB lasers for applications in networking, optical sensing, and power delivery.

## 1. Introduction

Among other microcavity lasers, the polymer distributed feedback (DFB) microcavity lasers have made several breakthroughs in light-based technologies due to the broad emission spectra, low threshold, small size, high optical efficiency, and simple manufacturing process [1,2,3,4]. Followed-up research advancements utilize a variety of fabrication techniques to generate DFB cavities, including interference lithography [5], nanoimprinting [6], direct writing [7], and electron beam lithography [8]. Compared with other methods, laser interference lithography is a low-cost and facile route to fabricate the grating, which could offer additional degrees of freedom for manipulating the performance of DFB polymer lasers. To date, some gain materials were suitable for DFB lasers, including polymers, dyes, perovskite, and colloidal quantum dots (CQDs) [9,10,11,12,13,14]. CQDs exhibit great potential as gain materials due to the excellent photoluminescence quantum yield (PLQY), great Stokes shift, widely tunable bandgaps, and low-cost effective chemical manufacturing [15,16,17,18,19]. The CQDs DFB lasers have a low threshold and stable performance [20].

Existing DFB polymer lasers have made considerable progress due to various manipulation techniques such as wavelength manipulation [21,22,23,24,25,26], threshold manipulation [27,28,29], intensity manipulation [30,31], and polarization manipulation [32,33,34]. But there are few studies about direction manipulation of DFB lasers [35] which are mainly about using the high-order DFB configuration to achieve the multi-direction emission of DFB lasers. However, the diffractive effect of the gratings characterizes the emission frequencies of the DFB lasers which in turn defines the lasing direction and puts limits on the design possibilities of the high-performance lasers. In addition, controlling the DFB lasing beam with a low divergence angle remains a challenge. Since the light confinement happens only along the direction that is orthogonal to grating lines, wave vector components of the optical mode in the grating-line direction are allowed inside the cavity, which results in the fan-shaped emission beam for 1D DFB lasers. While the 2D DFB structure exhibits cross-like and square-like lasing patterns due to vertical emission and diffraction at other angles respectively.

In this article, we fabricated second-order DFB lasers on optical fiber sidewall by using laser interference lithography. CQDs were used as gain material which was dip-coated on the grating. Emission spectra of this device are received from both the fiber facet and the vertical direction of the DFB structure. Compared with the vertical emission of the DFB lasers, the laser detected from the fiber facet has a smaller solid divergence angle owing to the confinement of the fiber waveguide effect. The combination of coupled wave theory with the multi-layered waveguide theory was employed to model the lasing performance of DFB fiber side-wall laser. The intensity of the coupled laser from the fiber facet is controlled by the coupling coefficient κ. The coupling coefficient is related to the angle between the grating vector and fiber axis, which can be explained by the coupled theory. In addition, by using a two-beam double exposure interference lithography, two juxtaposed parallel gratings with different periods were fabricated on an optical fiber sidewall. Therefore, we noted that cascading two different period grating structures on the fiber side-wall supports two-wavelength directional lasing with a considerably small solid divergence angle from the fiber facet. The proposed laser device combines the light confinement and guiding properties of optical fibers with the efficient optical feedback of DFB structure, achieving excellent self-aligned emission of multiple DFB lasers. With the help of the flexible fiber, the integration of multiple DFB lasers makes it very attractive for various potential applications such as high-performance light sources, highly sensitive sensors, long-term measurements or optical communication systems.

## 2. Design and Fabrication of Distributed Feedback Lasers on Optical Fiber Sidewall

**Figure 1:**
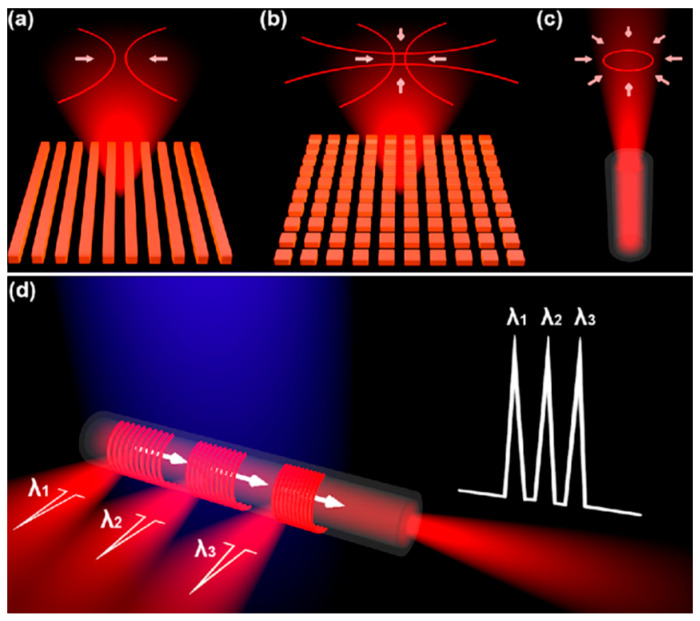
Schematic descriptions of the 1D and 2D DFB lasers on the fiber side-wall. (**a**) The lasing spot configuration from the surface-emitting of 1D DFB laser. (**b**) 2D surface-emitting DFB laser spot configuration. (**c**) Directional emission lasing spot configuration from the fiber facet. (**d**) Vertical emission and directional emission of multi-juxtaposed gratings with different periods on the fiber sidewall.

As shown in Figure 1a, the output beam of 1D DFB is two symmetric stripe-shape patterns that are parallel to the grating line. The light confinement only occurs along the direction which is orthogonal to the grating line.

The output beam of 2D DFB is diffracted from the sample in a cross-like profile as shown in Figure 1b. In this paper, we have proposed the integration of DFB laser configurations on the optical fiber sidewalls, whereas the coupling mechanism between the grating geometries and optical fiber exhibits directional DFB lasing from the fiber facet, as shown in Figure 1c. By assembling multi-juxtaposed gratings with different periods on the fiber sidewall, multi-wavelength emission spectra of this device are received from both the fiber facet and the vertical direction of the DFB structure, as shown in Figure 1d.

**Figure 2:**
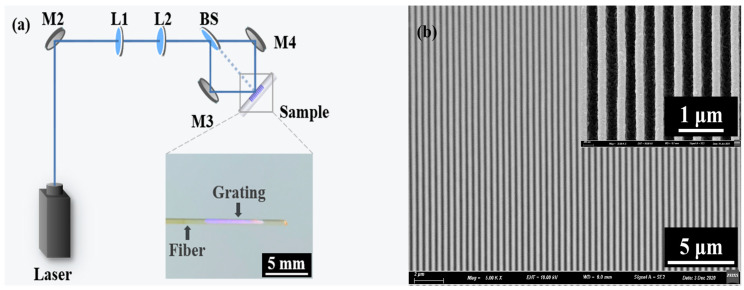
(**a**) Optical layout for the interference lithography technique. M1, M2, M3, and M4 represent reflectors. L1 and L2 represent optical lenses. BS represents beam splitter prism. The included angle between the two beams can be tuned to generate different grating periods for the respective interference fringes. The inset is the photograph of the DFB laser on the optical fiber sidewall. (**b**) Top-view scanning electron microscopy (SEM) image of the grating structure on the fiber sidewall. The inset is enlarged SEM images of the grating structure.

In this experiment, the CQDs DFB laser configurations include a quartz glass optical fiber as a substrate, integration of the second-order grating, and CQDs gain film. The polymer cladding of the fiber is removed using an acetone bath. The diameter of multimode optical fiber is 800 μm. The photo-resist (PR, AR-P3170, Strausberg, Germany) was dip-coated on the optical fiber sidewall, heated on the hot plate for 5 min at 110 °C. The optical layout of interference lithography is shown in Figure 2a. The proposed optical interference lithography layout comprises a diode-pumped solid-state laser (FLARE NX, Coherent, Santa Clara, CA, USA) with 343 nm, 550 Hz, and 1 ns for the exposure of PR films on the optical fiber up to 20 s, see Figure 2a. The grating period Λ of DFB lasers can be defined by the formula,

(1) $$\Lambda =\frac{\lambda }{2\sin\theta }$$

where λ is the wavelength of the ultraviolet laser and θ is half of the angle between two laser beams. Then put the exposed grating into the developer for 5 s. The inset of Figure 2a is the photograph of the sample, with an optical fiber diameter of 800 μm.

Figure 2b shows the top-view scanning electron microscopy (SEM, Hitachi S-4800, Hitachi, Tokyo, Japan) image of the grating structure on the fiber sidewall. It can be seen that the grating on the fiber sidewall can be fabricated very uniformly by this method. The inset of Figure 2b is an enlarged local structure of grating without gain film. The period of the grating is 390 nm, which can be calculated by Equation (1). The grating structure acts as the DFB cavity, which provides feedback for lasing. The CdSe/CdS/ZnS CQDs were dissolved into toluene with a concentration of 40 mg/mL, which was employed as the gain material. The solution of CQDs was dip-coated onto the grating structure, forming a CQDs DFB laser.

## 3. Results and Discussions

### 3.1. Experimental Results

**Figure 3:**
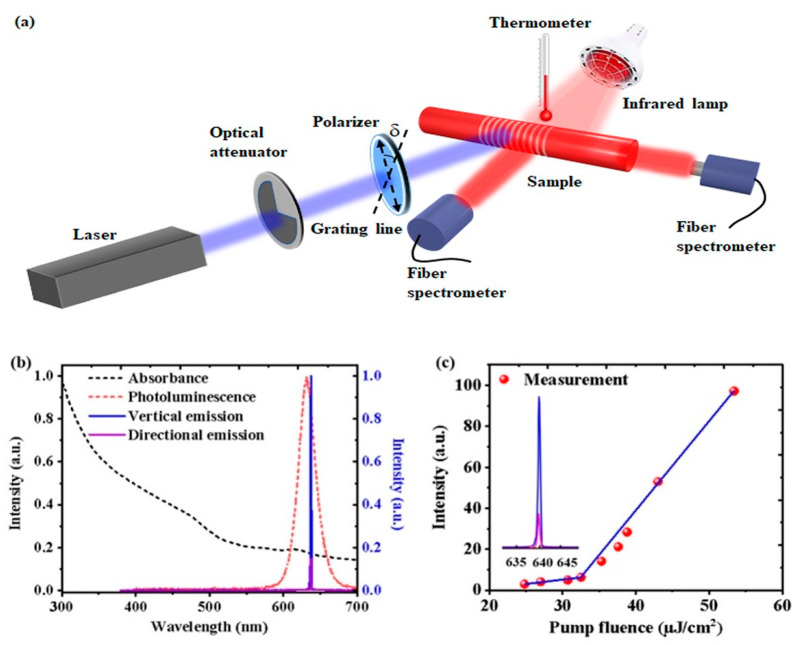
(**a**) Experimental diagram of lasing characterization from DFB lasers on fiber sidewall. δ is the angle between the grating line and the polarization direction of the pump beam. (**b**) The absorption (black dotted line), photoluminescence (red dotted line) of the CQDs, and emission spectra (solid lines) of the CQDs DFB lasers on fiber sidewall. (**c**) The threshold of the CQDs DFB lasers on fiber sidewall.

Figure 3a shows a characterization experimental diagram of CQDs DFB lasers on a fiber sidewall. In the experiment, the same laser used in the exposure process was employed as a pump source. A neutral density filter was used to adjust the pump power. A polarizer was inserted between the optical attenuator and the sample to change the pump polarization. δ is the angle between the grating line and the polarization direction of the pump beam. An infrared lamp and a thermometer were used to change and measure the temperature of the sample, respectively. It will be discussed in detail later. The emission spectra were measured by a spectrometer (Maya 2000 pro, Ocean Optics, Orlando, FL, USA). The emission spectra of this device are recorded as a surface-emitting DFB laser and directional lasing from the grating on the fiber sidewalls and fiber facet respectively.

The absorption (black dotted line), the photoluminescence (red dotted line) of the CQDs in toluene solution, and the emission spectra (solid lines) of the CQDs DFB lasers on fiber sidewall are shown in Figure 3b. CQDs have strong absorption of ultraviolet light, the photoluminescence (PL) peak is around 630 nm, the full width at half maximum (FWHM) is about 30 nm, and the fluorescence quantum efficiency (PLQY) is above 85 percent. Moreover, the CQDs have a great Stokes shift. Therefore, CQDs are ideal gain materials.

For a DFB polymer laser, the lasing wavelength λ satisfies the Bragg condition

(2) $$2n_{eff}\Lambda =m\lambda$$

where λ is the wavelength of the emission laser, Λ is the period of the grating, m is the diffraction order, and $n_{eff}$ is the index of the propagating mode and m is the Bragg order which equals 2 for the surface-emitting DFB laser. In this experiment, Λ is 390 nm and $n_{eff}$ is 1.634, therefore the lasing wavelength of the surface emission λ is 637.3 nm, as shown in Figure 3a. The threshold of the CQDs DFB lasers on fiber sidewall is 32.5 μJ/cm^2^ as shown in Figure 3c.

**Figure 4:**
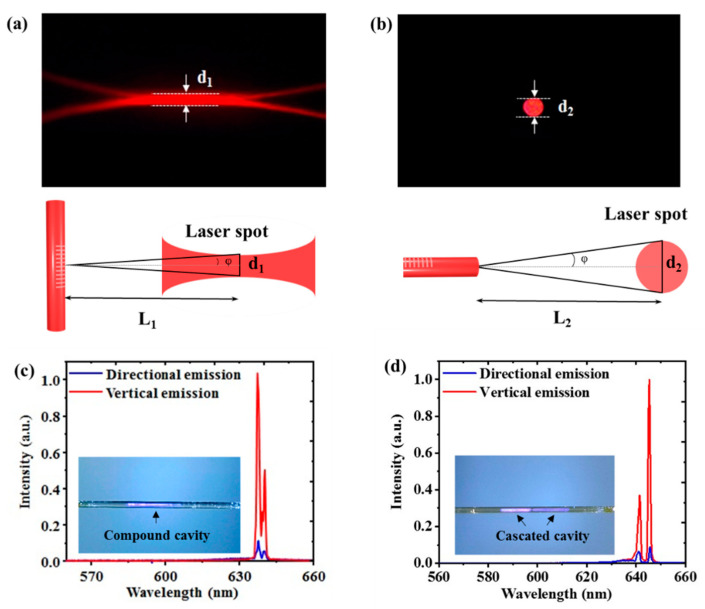
Photograph of the laser spot and schematic diagram for measuring divergence angle of (**a**) vertical emission of 1D DFB structure, and (**b**) directional emission from the fiber facet respectively. Comparison of the vertical emission (red curve) and the directional emission (blue curve) spectra from (**c**) the 2D compound DFB lasers, and (**d**) the cascaded DFB laser fabricated on fiber sidewall with different periods.

Figure 4a,b show the photograph of the laser spot of the vertical emission from the CQDs DFB lasers and the directional emission from the fiber facet, respectively. The divergence angle φ can be calculated by the formula,

(3) $$\tan\varphi =\frac{d}{2L}$$

where d is the width of the laser spot and L is the distance between the laser spot and the sample. The schematic diagrams for measuring the divergence angle of vertical emission and directional emission are shown in Figure 4a,b, respectively. The divergence angle φ of the laser spot is 17 mrad for vertical emission, and 34 mrad for directional emission. Although the divergence angle in the transverse direction of the directional emission is larger than that of vertical emission, the solid divergence angle of the directional emission is far less than that of vertical emission.

By using the two-step exposure produces, a 2D compound DFB cavity and a cascaded DFB cavity with different periods (Λ_1_ and Λ_2_) were fabricated on the optical fiber sidewall. The optical modes supported by the compound cavity or the cascaded cavity could be coupled into the waveguide mode of the optical fiber, and a two-wavelength spectrum could be received from both the fiber facet and the vertical direction of the DFB structure, as shown in Figure 4c,d. The inset of Figure 4c,d illustrates photographs of the compound cavity and the cascaded cavity on the fiber sidewall. The deep and light purple areas in the photograph of the cascaded cavity identify the two different grating periods on the fiber sidewall.

**Figure 5:**
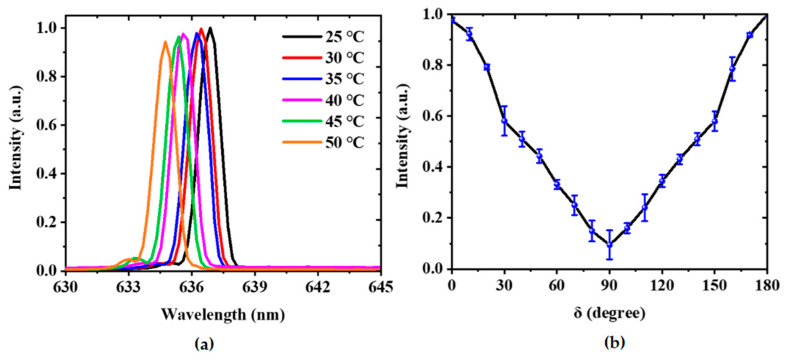
Influence of the temperature and the pump polarization on the laser performance. (**a**) Measured emission spectra at different ambient temperatures. (**b**) Output intensity as a function of the pump polarization.

To study the influence of the temperature and the pump polarization on the laser performance, an infrared lamp and a polarizer were introduced to the optical path in Figure 3a. Figure 5a shows the measured emission spectra at different ambient temperatures. It can be seen that the lasing wavelength changes slightly with the ambient temperature. The lasing wavelength shifts from 636.9 to 634.8 nm when the temperature changes from 25 to 50 °C. The variation of the lasing peak can be attributed to the thermo-optic effect of the polymer [36]. On the whole, the stability of the laser performance is acceptable for most potential applications. Figure 5b presents the relationship between the output intensity and the pump polarization. The maximum output intensity was achieved by adjusting the pump polarization parallel to the grating line. Note that the minimum output intensity was obtained by changing the pump polarization direction perpendicular to the grating line. Thus, the laser performance can be adjusted by the pump polarization.

### 3.2. Modeling

**Figure 6:**
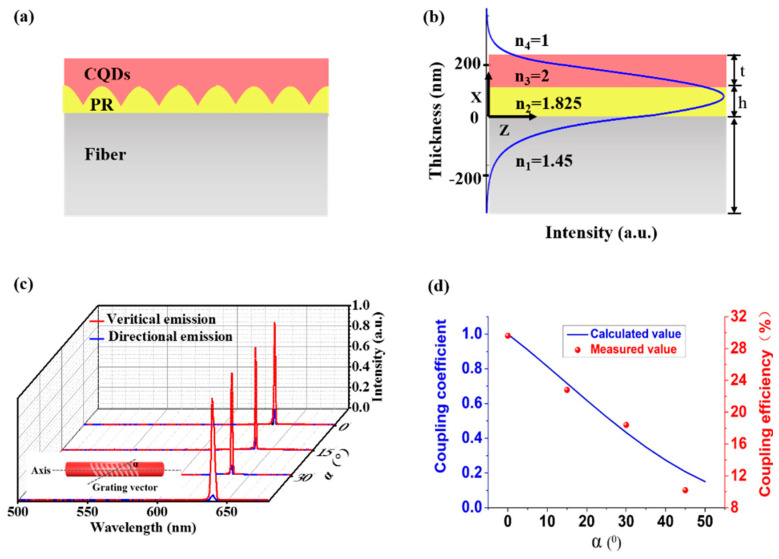
(**a**) Schematic cross-section through the CQDs DFB lasers structure on fiber sidewall. (**b**) Refractive index profile and mode profile at 640 nm for the same structure as in (**a**). (**c**) Emission spectra comparison of vertical emission (red curve) and the directional emission (blue curve). The intensity of the emission laser as a function of the angle between the grating vector and fiber axis—0 degrees, 15 degrees, 30 degrees, and 45 degrees, respectively. The inset defines the direction of the fiber axis and the grating vector. (**d**) The coupling coefficient as function of the angle between the grating vector and fiber axis.

The schematic description of the DFB lasers on the fiber sidewalls indicates that it can be regarded as a three-layered waveguide, see Figure 6a. The three-layered waveguide structure has an effective refractive index of 1.825 from the refractive index of CQDs (2), photoresist (1.45), and optical fiber (1.45). The thickness of the CQDs/grating/fiber layer was 0.12/0.12/800 μm. The multi-layered waveguide theory was employed to investigate the working mechanisms of DFB polymer lasers on the sidewalls of the optical fiber.

The field distribution of the mode in these four regions can be defined as below:

(4) $$E_{y}(x)=\left\{ \begin{array}{ll} a_{1}e^{b_{1}x}+a_{1}e^{-b_{1}x} & x\leq 0\\ a_{2}\sin\left(b_{2}x\right)+a_{2}\cos\left(b_{2}x\right) & 0<x\leq h\\ a_{3}e^{b_{3}x}+a_{3}e^{-b_{3}x} & h<x\leq h+t\\ a_{4}e^{-b_{4}x} & x>h+t \end{array}\right.$$

where $a_{i}$(i=1,2,3,4) represents the relative field amplitude coefficient, $b_{j}$(j=1,2,3,4) denotes the transverse wave number. $b_{1}=2\pi /\lambda \sqrt{n_{eff}^{2}-n_{1}^{2}}$, $b_{2}=2\pi /\lambda \sqrt{n_{2}^{2}-n_{eff}^{2}}$, $b_{3}=2\pi /\lambda \sqrt{n_{eff}^{2}-n_{3}^{2}}$, $b_{4}=2\pi /\lambda \sqrt{n_{eff}^{2}-n_{4}^{2}}$. By applying the boundary conditions, the distributions of the electric field in each guiding layer are obtained numerically by using MATLAB software.

The mode intensity distributions in the multilayered structure can be calculated based on the multi-layered waveguide theory, as indicated by the blue curve in Figure 6b. An optimized laser structure was attained that provides a well-balanced electric field distribution within the grating layer, gain layer, and optical fiber, as shown in Figure 6b. The lasing mode not only has to fulfill the Bragg condition of the grating but also to obey the resonant condition of the fiber waveguide.

The surface-emitting DFB lasing along the grating vector attained via second-order Bragg diffraction and it is normal to the sample surface, while the lasing supported by the waveguide mode was emitted from the fiber facet. The emission spectrum of vertical emission laser (red line) and the directional emission in fiber (blue line) as shown in Figure 6c. The coupling coefficient has related the angle between the grating vector and fiber axis. Therefore, the angle between the optical fiber axis and grating vector is modified from 0° to 45° (with 15° increments) to tune the coupling efficiency of the lasing into the fiber, which is denoted as the ratio of the intensity of directional emission to vertical emission. It can be noted that the increased angle between the optical fiber axis and grating vector decreased the coupling efficiency (29.6%, 22.8%, 18.4%, and 10.2%, respectively) of the lasing into the fiber, as depicted in Figure 6d.

The coupling effect can be described by the coupled-mode theory [37]. The resonant electric field of the DFB cavity and fiber waveguide could enter into the other’s field with a coupling constant κ. The coupling strength between the two modes is determined by the coupling coefficient κ, which was calculated by using the coupled-mode equation [38,39].

According to the coupled-wave theory, the coupled mode amplitudes can be expressed as

(5) $$\begin{array}{c} \frac{da_{1}}{dt}=-i\omega_{1}a_{1}+i\kappa a_{2}\\ \frac{da_{2}}{dt}=-i\omega_{2}a_{2}+i\kappa a_{1} \end{array}$$

where $a_{1}$ and $a_{2}$ are the mode intensity of DFB cavity and fiber waveguide respectively, and κ is the coupling coefficient.

The Fourier transform of a is:

(6) a(t)=∫A(ω)exp(−iωt)dω

Substituting Equation (6) into Equation (7), after arranging,

(7) $$\left|\begin{array}{cc} \sin\alpha \cdot \omega -\omega_{1} & -\kappa \\ -\kappa & (1+\cos\alpha )\cdot \omega -\omega_{2} \end{array}\right|=0$$

where $\omega_{1}$ and $\omega_{2}$ were the eigen-frequencies of DFB cavity and waveguide mode respectively, ω was the lasing frequency, and α was the angle between the grating vector and the fiber axis.

Equation (7) is deduced, the function between the angle α and the coupling coefficient κ is obtained:

(8) $$\kappa ={\left(\left(\omega_{1}-\omega \cdot \sin\alpha \right)\left(\omega_{2}-\omega \left(1+\cos\alpha \right)\right)\right)}^{\frac{1}{2}}$$

where $\omega_{1}$ and $\omega_{2}$ were the eigen-frequencies of DFB cavity and waveguide mode respectively, ω was the lasing frequency, and α was the angle between the grating vector and the fiber axis. The coupling coefficient κ can be adjusted by changing the angle α as shown in Equation (8). Figure 6d showed that the coupling coefficient decreased with increasing α and the experimental results agree with the theoretical model. When α=0 the two resonant modes overlap along the axis of the fiber to the maximum extent. So, when the angle between the grating vector and fiber axis is 0 degrees, the coupling efficiency is the highest, which is 29.6%. When the angle is more than 45 degrees, the CQDs DFB laser is difficult to be excited, which may be caused by the loss of mismatch of the two modes.

## 4. Conclusions

A CQDs DFB laser on optical fiber sidewall was fabricated by combining interference lithography and dip-coating method. The grating parameters can be adjusted simply by changing the angle between two interference beams. The number of CQDs DFB lasers can be controlled by multiple exposures. We proposed a simple and low-cost fabrication technique to fabricate CQDs DFB lasers on optical fiber sidewall. Due to the coupling effect, the directional emission with a small divergence angle can be obtained from the fiber facet, and the maximum coupling efficiency is 29.6%. The coupling coefficient decreased with the increasing angle between the grating vector and fiber axis, and the experimental results agree with the theoretical model. This laser device combines the light confinement and guiding properties of optical fibers with the efficient optical feedback of DFB structure, providing monochromatic and polychromatic self-aligned emission of distributed feedback lasers, which are crucial for optical information processing in functional, optical, and electronic nanoscale circuits.

## Data Availability

Data sharing is not available.
